# Case Report: Durable Response to Very Low Dose Tyrosine Kinase Inhibitors in Advanced Hepatocellular Carcinoma

**DOI:** 10.3389/fonc.2021.780798

**Published:** 2021-12-16

**Authors:** Tin-Yun Tang, Katherine Daunov, Richard T. Lee

**Affiliations:** ^1^ Department of Medicine, University Hospitals Cleveland Medical Center, Cleveland, OH, United States; ^2^ Division of Hematology and Oncology, University Hospitals Seidman Cancer Center, Cleveland, OH, United States; ^3^ Case Western Reserve University School of Medicine, Cleveland, OH, United States

**Keywords:** hepatocellular carcinoma (HCC), sorafenib, regorafenib, cabozantinib, low dose, minimal effective dose

## Abstract

The oral tyrosine kinase inhibitors (TKI) sorafenib, regorafenib, and cabozantinib are approved for advanced hepatocellular carcinoma (aHCC) and improve survival. However, patients on these medications frequently require dose reductions or discontinuation due to multiple side effects leading to poor tolerability. Here we report three different aHCC patients with clinical responses outlasting those reported in their corresponding Phase 3 clinical trials on 1/8th the target dose for sorafenib, 1/4th the target dose for regorafenib and 1/6th the target dose for cabozantinib respectively. As these doses are below the minimal recommended doses on the FDA labels, this case series provides a preliminary demonstration that low dose TKI therapy can be effective and patients on TKIs should first assess for clinical response before empirically discontinuing TKI therapy on the basis of tolerating only a low dose.

## Introduction

The oral tyrosine kinase inhibitors (TKI) sorafenib, regorafenib, and cabozantinib are approved for advanced hepatocellular carcinoma (aHCC) and improve overall survival (OS). These are prescribed at target doses of 400mg twice daily (BID), 160mg daily (QD) and 60mg QD respectively under a maximum tolerated dose (MTD) paradigm and are associated with multiple toxicities that often require dose reductions. The prognosis for patients with aHCC is poor and new medications have been challenging to discover – sorafenib was established and remains a first-line therapy for aHCC based upon a 2-3 month OS increase against placebo (1, 2). The vascular endothelial growth factor receptor 2 (VEGFR2) inhibitor ramucirumab is the only non-TKI treatment FDA approved for aHCC, demonstrating how challenging developing new therapeutic approaches outside of TKI therapy has been (3, 4). In regards to immunotherapy, only within the last year has atezolizumab plus bevacizumab become standard of care first line therapy based on the results of the IMbrave150 trial with a progression free survival of 6.8 months, and nivolumab and pembroluzimab are approved for second line therapy (5).

TKIs remain the mainstay therapeutic approach for patients that cannot tolerate or progress on atezolizumab plus bevacizumab. Multiple attempts have been made with limited success to elucidate potential prognostic factors for TKI therapy with biomarkers such as alpha-fetoprotein (AFP), angio-potietin-2 (Ang-2), vascular endothelial growth factor-A (VEGF-A) or clinical characteristics such as viral hepatitis status, diabetes and dermatologic toxicities (6). Since there are no well validated predictive markers for response to TKI therapy, when to adhere to, discontinue, or switch TKI agents when patients report severe side effects is clinically subjective. The current standard practice is to discontinue a particular TKI agent and pursue a different line of therapy if patients are unable to tolerate minimum target doses under the assumption that lower doses of TKIs are subtherapeutic. Discontinuation occurs even prior to or without response evaluation despite few clinical studies having examined the minimal effective dose (MED) for TKIs, which have noncytotoxic mechanism of actions. Our case report demonstrates that patients with advanced hepatocellular carcinoma (aHCC) should consider low dose TKI therapy (below FDA guidelines) and assess for clinical response prior to discontinuation of therapy.

## Case Description

Patient 1 had Hepatitis C related cirrhosis and was diagnosed at age 70 with Stage IV HCC and underwent multiple rounds of locoregional therapy before progression of disease. Alpha-fetoprotein (AFP) before initiating sorafenib was 39,478 ng/mL (ref. 0-9 ng/mL) and was Child-Pugh score A. The patient started sorafenib therapy at 200mg QD and titrated up to 200mg BID; he tolerated 200mg BID for 1 week before reducing back to 200mg QD due to severe hand foot skin reaction (HFSR). Multiple attempts to titrate up from 200mg QD failed due to ongoing HFSR and fatigue. An abridged cycle of 200mg QD for 2 weeks on and off was unsuccessful due to intolerance to daily dosing. The patient arrived at a steady dose of 200mg every other day (QOD) and AFP decreased to 2ng/mL at 4 months following initiation of sorafenib with sustained response. At time of writing, progression free survival (PFS) is 36 months and the patient continues taking sorafenib 200mg QOD.

Patient 2 had both alcohol related and Hepatitis C related cirrhosis and was diagnosed at age 63 with HCC and underwent 2 rounds of locoregional therapy with TACE before progression to multifocal bilobar disease. He was treated with sorafenib for 3 months before discontinuation of therapy due to disease progression. Regorafenib was subsequently started at 80mg QD with a plan for weekly dose increases of 40mg/day to the label dosage of 160mg QD for the first 21 days of a 28-day cycle. He was Child-Pugh score A at this point in time. The patient developed anorexia, severe HFSR and hypertension at 120mg AD and regorafenib was restarted at 40mg qd. A repeat attempt at 80mg resulted in HFSR, mouth sores and subjective cognitive changes. Patient arrived at a stable dose regiment of 40mg QD. Time to Radiologic Progression (TTP) was 32 months and in the last 12 months was on an even lower dose at 40mg QOD before progression of disease and discontinuation of regorafenib. OS was 37 months.

Patient 3 was diagnosed at age 66 with Stage II HCC. Due to underlying both alcoholic and Hepatitis C related cirrhosis and portal vein thrombosis the patient was not a candidate for tumor directed therapy and instead initiated on sorafenib. Sorafenib treatment occurred for 1 month at a low dose of 200mg QD before it was permanently discontinued due to severe drug induced liver injury. Nivolumab was started as second line therapy but stopped after 3 cycles due to multiple immune-related adverse events including pneumonitis, dermatitis, stomatitis, and nephritis. Following further progression of disease, patient was started on regorafenib which he tolerated at 80mg QD for 3 months before it was also discontinued due to severe gastrointestinal hemorrhage. Cabozantinib treatment was then started, but at a low dose of 20mg QD given the multitude of prior therapy related adverse events. He was Child-Pugh score B at this point in time. Subsequent development of severe HFSR at 60mg QD to the point it limited mobility has ultimately resulted in dose reductions to a steady dose of 20mg QOD. The patient at current time has PFS of 13 months dated from cabozantinib initiation.

**Figure 1:**
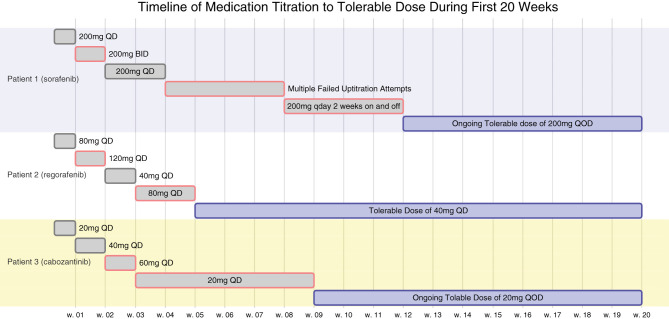
All 3 patients underwent multiple attempts at titrating their respective TKI therapies to the FDA label dosages but were limited by toxicities. Their titration timeline is as above. At 6 weeks, none of the patients would have met their corresponding minimum FDA label dosages of sorafenib 400mg QOD, regorafenib 80mg QD, or cabozantinib 20mg QD.

**Figure 1** is a timeline of the patient’s titration dosages as described above.

Written informed consent for publication of the above case histories were obtained from the patient or next of kin. The University Hospitals institutional review board (IRB) has determined that publication of the above patient cases does not require IRB review or approval.

## Discussion

The SHARP, RESORCE and CELESTIAL trials that led to FDA approval of sorafenib, regorafenib and cabonzantinib in aHCC at the respective maximal tolerated dose (MTD) targets of 400mg BID 160mg QD and 60mg QD allowed up to 2 dose reductions (lowest doses 400mg QOD, 80mg QD, and 20mg QD respectively) before treatment was discontinued, reported to be 38%, 25% and 16%, respectively (1, 7, 8). This practice has carried over to clinical practice despite the lack of studies on minimal effective dosing (MED) for TKIs which have a cytostatic mechanism of action and therefore do not lend themselves to a MTD paradigm (9–11).

**Figure 2:**
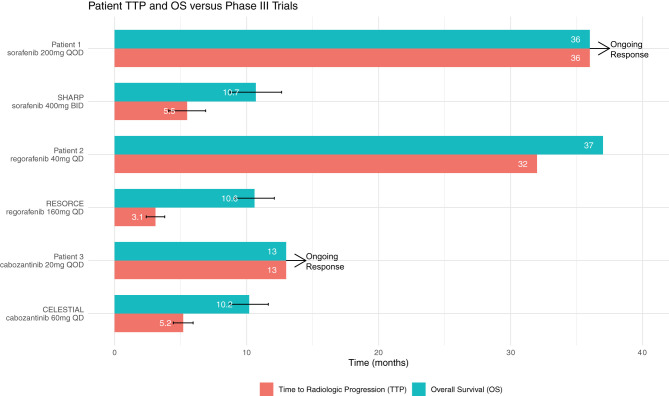
The average Time to Radiologic Progression (TTP) and Overall Survival (OS) as reported in the SHARP, RESORCE and CELESTIAL trials are above. Patient 1’s and Patient 2’s corresponding TTP and OS on TKI therapy are both significantly longer than the trial averages despite being on much lower doses of therapy than the trial regiments. Patient 3 continues to have PFS beyond the CELESTIAL reported TTP on 1/6^th^ the clinical trial target dose.

Had Patients 1 or 2 been clinical trial participants or followed an empirical discontinuation framework based on dosing, they would have discontinued TKI therapy prior to the initial 6-week response evaluation (**Figure 1**). Patient 1 continues to experience ongoing progression free survival (PFS) on 1/8th the normal targeted dose for sorafenib and Patient 2 also experienced prolonged PFS and overall survival (OS) on 1/4th to 1/8th the label dose for regorafenib (**Table 1**). These patients’ clinical responses far exceed those demonstrated in the SHARP or RESORCE trials (**Figure 2**). Although the CELESTIAL trial did not explicitly remove patients based on a minimal dose, the current FDA label indication continues to recommend discontinuation of therapy below 20mg QD. Patient 3 was unable to tolerate multiple different lines of therapy and despite being on 1/2 the minimal recommended dose, continues to have PFS beyond the CELESTIAL reported PFS and OS (**Figure 2**). Had TKI therapy been discontinued solely on the inability to tolerate the recommended target doses on all three of these patients, they would have missed significant therapeutic opportunities for prolonged disease control. As an added benefit, all three of the patients expressed improvement in their overall quality of life when their TKI’s were at easier tolerated dosages.

**Table 1:** Dosing regiments for sorafenib, regorafenib, and cabozantinib.

	Dose	Frequency	Reduction
**sorafenib**			
Patient 1	200	QOD	
SHARP	400	BID	
	400	QD	1^st^
	400	qod	2^nd^
**regorafenib**			
Patient 2	40	QD	
RESORCE	160	QD	
	120	QD	1^st^
	80	QD	2^nd^
**cabozantinib**			
Patient 3	20	QOD	
CELESTIAL	60	QD	
	40	QD	1^st^
	20	QD	2^nd^

QOD, every other day; BID, twice daily; QD, daily.

All three of the above described patients had HFSR as a dose limiting toxicity for their corresponding TKIs, which is particularly notable as there is a growing body of literature correlating the early development of HFSR with positive response to therapy. This phenomena has been thoroughly reviewed elsewhere (12). Dedicated studies with larger cohorts closely examining the timing of onset for HFSR in conjunction with usage of low dose TKI’s could provide further clinical information clarifying this association’s prognostic value. At current, the PROSASH-II score is the only validated prognostic tool in the context of sorafenib response and does not include timing of onset for HFSR (13).

Dosing regiments in clinical trials are generally designed with the MTD paradigms that originated with traditional cytotoxic chemotherapies wherein higher doses display increased levels of efficacy but are limited by dose limiting toxicities. Patients often require time to recover from dose limiting toxicities and it is for this reason that MTD paradigms frequently incorporate 7 days off therapy in a 28 day cycle. MED and other synonymous concepts such as optimal biological dosing and biological efficacious dosing as alternative paradigms have started to garner increasing attention as more noncytotoxic chemotherapies have become clinically available and mechanistically do not always increase in efficacy with dosage (14, 15). There has been growing body of literature in recent years supporting that clinical outcomes can be improved by shifting away from MTD treatment paradigms. For example, two studies with sorafenib have demonstrated that allowing for a higher cumulative dose over a treatment lifetime through careful dose adjustments are independent predictors of survival and demonstrate increases in progression free survival and overall survival (16, 17). Additionally, the related paradigm of metronomic chemotherapy regiments wherein patient are dosed intermittently or even continuously at lower doses is a promising application of a MED based paradigm and early successes in HCC with capecitabine in have been reported (18–20).

These cases serve as preliminary clinical observations that aHCC patients can have prolonged clinical responses to doses of TKIs below the approved FDA label doses. Given the lack of biomarkers that predict therapeutic response to TKIs, we believe our patients suggest that there could be utility to evaluating potential response to therapy before discontinuing TKI therapy. To our knowledge, there are no prior reports describing the clinical outcomes of aHCC patients on very low dose TKIs. The therapeutic benefit seen in the reported patients could potentially justify further translational research or observational studies.

An important limitation to acknowledge is that we report here on only 3 patients; it is premature to recommend routine use of TKIs at low doses based on the reported patients and more research work is necessary. Future directions for translational studies could for example be evaluating whether TKIs at low dose have a therapeutic mechanism of action separate from kinase inhibition. For example, sorafenib at 1/10^th^ the target dose has very recently been demonstrated to act as a mitochondrial uncoupler and through that mechanism of action delay progression of non-alcoholic steatohepatitis (21). It could be intriguing to see if this mechanism of action extends to other TKIs such as regorafenib and cabozantinib. From a clinical perspective, perhaps patients with unusually strong HFSR, such as that seen in the reported patients, could be an appropriate subset of patients that could be enrolled in a clinical trial for low dose usage of TKIs. Prior studies have detected a correlation with HFSR and better outcomes with sorafenib (22). Determining whether this is a TKI class effect or idiosyncratic to sorafenib would be clinically useful.

Notably, TKI therapy is indicated in multiple other cancers, including common ones such as colorectal cancer and the above proposed future research directions could perhaps also include these indications. In conclusion, here we report on 3 cases of prolonged responses in aHCC patients to low doses of 3 different TKIs which hints at a broader dosing paradigm for non-cytotoxic therapies with unknown MEDs and have implications in both clinical practice as well as clinical trial design.

## Data Availability Statement

The original contributions presented in the study are included in the article/supplementary material. Further inquiries can be directed to the corresponding author.

## Ethics Statement

The studies involving human participants were reviewed and approved by University Hospitals IRB. The patients/participants provided their written informed consent to participate in this study. Written informed consent was obtained from the individual(s) for the publication of any potentially identifiable images or data included in this article.

## Author Contributions

TT, KD, and RL contributed to the conception and design of the study. TT wrote the first draft of the manuscript. All authors contributed to manuscript revision, read, and approved the submitted version.

## Conflict of Interest

The authors declare that the research was conducted in the absence of any commercial or financial relationships that could be construed as a potential conflict of interest.

## Publisher’s Note

All claims expressed in this article are solely those of the authors and do not necessarily represent those of their affiliated organizations, or those of the publisher, the editors and the reviewers. Any product that may be evaluated in this article, or claim that may be made by its manufacturer, is not guaranteed or endorsed by the publisher.
